# Analysis of Environmental Variables and Carbon Input on Soil Microbiome, Metabolome and Disease Control Efficacy in Strawberry Attributable to Anaerobic Soil Disinfestation

**DOI:** 10.3390/microorganisms9081638

**Published:** 2021-07-31

**Authors:** Shashika S. Hewavitharana, Emmi Klarer, Joji Muramoto, Carol Shennan, Mark Mazzola

**Affiliations:** 1 Horticulture and Crop Science Department and Strawberry Center, California Polytechnic State University, San Luis Obispo, CA 93407, USA; 2 Department of Plant Pathology, Washington State University, Pullman, WA 99164, USA; emmi.klarer@usda.gov; 3 Department of Environmental Studies, University of California, Santa Cruz, 1156 High Street, Santa Cruz, CA 95064, USA; joji@ucsc.edu (J.M.); cshennan@ucsc.edu (C.S.); 4 United States Department of Agriculture, Agricultural Research Service, Wenatchee, WA 98801, USA; 5 Department of Plant Pathology, Stellenbosch University, Matieland 7600, South Africa

**Keywords:** strawberry diseases, Macrophomina crown rot, Fusarium wilt, reductive soil disinfestation, biological soil disinfestation, incubation temperature, incubation duration

## Abstract

Charcoal rot and Fusarium wilt, caused by *Macrophomina phaseolina* and *Fusarium oxysporum* f. sp. *fragariae*, respectively, are major soil-borne diseases of strawberry that have caused significant crop losses in California. Anaerobic soil disinfestation has been studied as an industry-level option to replace soil fumigants to manage these serious diseases. Studies were conducted to discern whether Gramineae carbon input type, incubation temperature, or incubation duration influences the efficacy of this disease control tactic. In experiments conducted using ‘low rate’ amendment applications at moderate day/night temperatures (24/18 °C), and carbon inputs (orchard grass, wheat, and rice bran) induced an initial proliferation and subsequent decline in soil density of the Fusarium wilt pathogen. This trend coincided with the onset of anaerobic conditions and a corresponding generation of various anti-fungal compounds, including volatile organic acids, hydrocarbons, and sulfur compounds. Generation of these metabolites was associated with increases in populations of *Clostridium* spp. Overall, carbon input and incubation temperature, but not incubation duration, significantly influenced disease suppression. All Gramineae carbon inputs altered the soil microbiome and metabolome in a similar fashion, though the timing and maximum yield of specific metabolites varied with input type. Fusarium wilt and charcoal rot suppression were superior when anaerobic soil disinfestation was conducted using standard amendment rates of 20 t ha^−1^ at elevated temperatures combined with a 3-week incubation period. Findings indicate that anaerobic soil disinfestation can be further optimized by modulating carbon source and incubation temperature, allowing the maximum generation of antifungal toxic volatile compounds. Outcomes also indicate that carbon input and environmental variables may influence treatment efficacy in a target pathogen-dependent manner which will require pathogen-specific optimization of treatment protocols.

## 1. Introduction

The United States is the world’s largest producer of strawberries (*Fragaria* × *ananassa* Duchesne), with California accounting for 92% of total U.S. production [1]. California also produces approximately one billion strawberry transplants annually that are shipped worldwide and must meet stringent phytosanitary standards [2]. Although strawberry is a high-value crop, its production is limited by numerous pest problems, including a diversity of lethal soil-borne diseases such as Verticillium wilt, Fusarium wilt, Macrophomina crown rot, and Phytophthora crown rot [3]. Historically, in California, strawberry production was designed to utilize soil fumigation as an integral component of the cropping system and circumvent the potential for economic damage resulting from such disease occurrence [4]. Soil-borne diseases are estimated to reduce strawberry yields by 20–30% in the absence of pre-plant soil fumigation [5,6]. While highly effective, soil fumigation has increasingly been subjected to regulatory restrictions or outright bans as a management practice due to potential environmental and human health implications [7]. Loss of the widely used and highly efficacious soil fumigant methyl bromide due to regulatory action has had significant ramifications for the economic vitality of this industry [3].

Following the ban on methyl bromide, a diversity of alternative fumigant chemistries was evaluated for disease control capability in the California strawberry production system [8]. In initial trials, certain alternative fumigants were found to be effective methyl bromide replacements for soil-borne disease control in both strawberry fruit and nursery plant production systems. However, the economic analysis indicated that methyl bromide/chloropicrin was the most cost-effective treatment [8,9]. The elimination of methyl bromide and adoption of alternative fumigants corresponded with increases in crop losses from emerging diseases caused by *Fusarium oxysporum* f. sp. *fragariae* [10] and *Macrophomina phaseolina* [11,12]. Subsequent trials have demonstrated that currently available fumigant chemistries provide insufficient control of both of these fungal pathogens over the course of the California strawberry production season [13].

Anaerobic soil disinfestation (ASD) is a soil-borne management practice that involves three steps: (i) incorporation of a labile carbon source, (ii) inundation to fill pore-spaces in soil, and (iii) covering the soil with an oxygen impermeable film, and incubating for several weeks [14]. This technique has been examined as a potential alternative to fumigation for soil-borne disease control in numerous cropping systems [15], including strawberry [16,17]. Disease control attained in response to ASD is thought to be derived primarily through the production of various active chemistries, such as volatile fatty acids, methyl sulfide compounds, and hydrocarbons possessing antimicrobial activity [18,19,20]. ASD yields consistent dynamic changes in the composition of the soil microbiome associated with disease control [20,21,22]. In general, it is believed that the action of the altered microbiome in disease control is through amendment-driven transformation of the soil metabolome, resulting from community metabolism, rather than direct parasitism or antibiotic activity toward target pathogens [20].

Disease control efficacy in response to ASD is influenced by numerous factors, including soil temperature, C:N ratio of the organic input, quantity of amendment applied, soil moisture content, and the specific pathogen targeted [23,24]. Although ASD has consistently provided control of Verticillium wilt [25], it has provided inconsistent or ineffective control of *F. oxysporum* f. sp. *fragariae*, the cause of Fusarium wilt [26,27], and *M. phaseolina,* which incites charcoal rot, in California strawberry field trials [16,27]. Significant effort has been targeted toward enhancing ASD efficacy for soil-borne disease control by manipulating application protocols or environmental parameters that may influence the production of potential active chemistries. Such variables include carbon source input, irrigation quantity and duration, incubation duration, tarping system, and soil temperature [23,24].

Several studies have provided insight into the significant role of carbon source input type on the generation of active metabolites during the ASD process. However, comprehensive comparative analyses of changes to the microbiome, metabolome, and consequent outcomes in determining disease control efficacy are warranted. The objectives of this study were to evaluate: (i) the effect of incubation temperature, (ii) duration of the incubation period, and (iii) carbon source on the soil metabolome, microbiome and efficacy of ASD for the control of *F. oxysporum* f. sp. *fragariae*, and *M. phaseolina*.

## 2. Materials and Methods

### 2.1. Soils

Studies were conducted either in soils naturally infested with the target pathogen *F. oxysporum* f. sp. *fragariae* or *M. phaseolina,* or soil was artificially infested with the pathogen, as described in Table 1. Naturally infested soils were sourced from strawberry fields in Watsonville, California, and soils used for artificial infestation were obtained from sites in Orondo and Rock Island, Washington, with no previous history of strawberry cultivation.

### 2.2. Preparation of Pathogen Inoculum and Soil Infestation

Inoculum of *F. oxysporum* f. sp. *fragariae* isolate 62-1230 and *M. phaseolina* isolate MAC 07-03 were prepared as described below. Cultures of *F. oxysporum* f. sp. *fragariae* were grown on 1/10th-strength potato dextrose agar for two weeks under ambient conditions. Agar plugs 1 cm in diameter were transferred from the actively growing edge of the fungal culture to 100 mL of 1/10th-strength potato dextrose broth and grown for two weeks under ambient conditions on a platform shaker at 100 rpm. The inoculum broth was strained through sterile cheesecloth and centrifuged at 4400 rpm for 10 min. Pelleted conidia were collected by removing the supernatant and washed by refilling the tubes with sterile distilled water and centrifuging at 4400 rpm for 10 min. Conidia were washed twice, at which point the water supernatant was removed, and the pellets re-suspended in sterile distilled water.

*Macrophomina phaseolina* isolate MAC 07-03 was grown in 20 mL of potato dextrose broth amended with ampicillin (100 μg mL^−1^) in Petri plates. Cultures were grown for one month at 30 °C. Mycelial mats with microsclerotia were removed from eight Petri plates with sterile forceps, dispensed into a blender, and the culture was comminuted by blending for 30 s in 750 mL sterile distilled water. The resulting suspension was poured in 25 mL aliquots into 50 mL sterile Falcon tubes, and 15 mL of sterile distilled water was added. The suspension was then vortexed briefly prior to allowing microsclerotia to settle at the bottom of the tube. The supernatant was decanted, 40 mL distilled water was added, and vortexed briefly and microsclerotia were allowed to sediment for 10 min. This process was repeated until a negligible quantity of mycelium was observed in a 10 μL subsample by microscopic examination at 20 X.

With the exception of the ASD incubation temperature and duration study conducted with naturally infested soil (Table 1), other soils were artificially infested with one of the two target pathogens. Those soils artificially inoculated with a pathogen had not previously been planted with strawberry. Hence, it was assumed that these soils had little to no natural inoculum of *F. oxysporum* f. sp. *fragariae* and *M. phaseolina*. The conidial suspension of *F. oxysporum* f. sp. *fragariae* was applied to soil to attain an inoculum density of 200–300 colony forming units (CFU) g^−1^ soil. The suspension of *M. phaseolina* microsclerotia was applied to soil to attain an inoculum density of 2.5 microsclerotia g^−1^ soil reflecting the thresholds inferred for the diseases [28].

### 2.3. Pathogen Quantification in Soil

The soil density of *M. phaesolina* was determined by real-time quantitative PCR (qPCR). Soil samples (25 g) were obtained from each biological replicate immediately after completing the anaerobic phase of incubation and each sampling time point thereafter. DNA was extracted from two 0.25 g sub-samples using the PowerSoil^®^ DNA isolation kit (MO BIO Laboratories Inc., Carlsbad, CA). For quantification of *M. phaseolina*, qPCR employed the primer pair MpKFI (5′-CCG CCA GAG GAC TAT CAA AC-3′ [29]/MpKRI (5′-GCT CCG AAG CGA GGT GTA TT-3′) [30] and was conducted using the reaction mixture and amplification conditions described by [30]. For *F. oxysporum*, soil density was determined using culture-based methods, as described in [31], or qPCR using sampling and DNA extraction as noted above. qPCR was conducted using the primer pair PFO2 (5′-CCA GGT ATT ACA CGG T-3′)/PFO3 (5′-CGG GGG ATA AAG GCG G-3′) [32]. Each 10 µL amplification reaction mixture consisted of 1 µL of a 1:10 dilution of soil DNA extract, 0.1 µL of each primer, 3.0 µL SYBR Green PCR Master Mix (Applied Biosystems, Warrington, United Kingdom), and 5.8 µL nuclease-free water. Standard curves were prepared using DNA extracted from *F. oxysporum* f. sp. *fragariae* isolate 62-1230 with a 10-fold dilution ranging from 1000 pg µL^−1^ to 0.01 pg µL^−1^. Each sample, standard curve concentration, and no template control had three technical replicates of identical reaction mixtures and volumes. Amplification was performed under the following cycling parameters: 95 °C for 10 min; 40 cycles of 95 °C for 30 s, 63 °C for 30 s, and 72 °C for 30 s; 95 °C for 15 s; 60 °C for 1 min, and 95 °C for 15 s, respectively.

### 2.4. Carbon Sources

Three carbon inputs were evaluated independently at the rates specified in Table 1: ground orchard grass (GR; *Dactylis glomerata* L.); ground wheatgrass (WH; *Triticum aestivum* L. cv. ‘Alpowa’); and rice bran (RB; *Oryza sativa* L., Farm Fuel Inc. LLC, Santa Cruz, CA, USA). The ‘standard rate’ of 20 t ha^−1^ corresponds with the rate previously employed in a multitude of controlled environments and field studies [17,23]. Multiple trials employed a ‘low rate’ amendment treatment due to the previously observed amplification of *F. oxysporum* f. sp. *fragariae* in response to ASD carbon inputs at the ‘standard rate’, as discussed below. All three amendments had a particle size of 0.5 to 1.0 mm. Estimated nutrient concentration of RB was N = 2.49%, P = 1.53%, K = 1.60%, S =0.18%, C:N ratio = 19.1, and pH = 6.2 (Soiltest Farm Consultants, Inc., Moses Lake, WA, USA). For GR, estimated nutrient composition and pH of the grass residues was N = 2.19%, P = 0.24%, K= 1.73% and S = 0.18%; a C:N ratio of 19:1 and pH of 6.3. The estimated composition of WH was N = 3.28%, P = 0.52%, K= 1.07% and S = 0.46%; a C:N ratio of 14:1 and pH of 5.7 (Soiltest Farm Consultants, Inc.).

### 2.5. Application of ASD treatments

#### 2.5.1. ASD Treatment General Method

The ASD treatment for all experiments was conducted as described in [19,20]. Briefly, the specific carbon inputs noted in Table 1 were evenly mixed into soils. Orchard soils were placed in 12-cm-diameter, 1180 mL plastic pots (McConkey Co., Sumner, WA, USA). The soils were irrigated with distilled water to field capacity. The potted soil container was enclosed in two gas-impermeable Bitran^®^ bags (Com–Pac International, Carbondale, IL, USA), resulting in an airtight container. The experimental units were incubated at the temperatures specified in Table 1. ASD was conducted over a 3-week period, and soils were aerated for 2 weeks prior to planting with strawberry.

#### 2.5.2. Specific Experimental Designs

##### ASD Incubation Temperature and Duration

The experiment was conducted in soil naturally infested with *F. oxysporum* f. sp. *fragariae* (trials 1 and 2; Table 1). Trials 1 and 2 employed three factors including day/night soil temperature regime [low(L)-16/10 °C; medium low (ML)₋24/18 °C; medium M)-32/26 °C; high (H)-40/34 °C], ‘standard rate’ carbon amendment [RB or no-amendment non-pasteurized aerobic control (NTC), and incubation period duration [3 weeks or 6 weeks]. The experiment utilized a completely randomized block design with four replicates.

##### Gramineae Carbon Source effect on ASD Efficacy

ASD treatments using the ‘standard rate’ of GR, RB, or WH were applied to *M. phaseolina* artificially infested soil. The trial included a no-treatment aerobic control using non-pasteurized soil (NTC) and pasteurized controls (PC) in a completely randomized design with ten replicates. Pasteurized controls consisted of soil heated to 70 °C for 8 h on two successive days, with 24 h in between each heating session. The effect of volatiles generated during ASD on in vitro growth of *M. phaseolina* isolate MAC 07-03 was conducted using previously described methods [19]. The diameter of the *M. phaseolina* cultures exposed to volatiles generated during ASD was measured at two perpendicular axes. At the completion of the ASD period, a mycelial plug was removed from the growing margin of the culture, transferred onto fresh 1/10th-strength PDA amended with kanamycin (75 μg mL^−1^), and streptomycin (100 μg mL^−1^). The colony diameter was determined after a 3-day incubation at 24/18 °C day/night temperature regime with a 12 h photoperiod.

As carbon source inputs were shown to elevate soil populations of *F. oxysporum* f. sp. *fragariae* [20,24,33], trials sought to assess whether effective disease control could be attained at lower input rates (Table 1) and minimize amplification of *F. oxysporum* f. sp. *fragariae* soil populations. A trial (‘low rate’ trial 1) was conducted to evaluate ASD treatments using ‘low rate’ of GR or RB as the carbon input in *F. oxysporum* f. sp. *fragariae* infested soil. The trial included a no-treatment aerobic control (NTC) and used a completely randomized design with eight biological replicates, totaling 24 experimental units. After soil incorporation of the amendment, ASD incubation was conducted over a 21-day period in an environmental growth chamber at 24/18 °C day/night temperature regime with a 12 h photoperiod. The soil density of *F. oxysporum* f. sp. *fragariae* was determined at day 0 and day 21.

An additional ‘low rate’ carbon input ASD trial (‘low rate’ trial 2) included ASD treatments of GR, RB, and WH, and a no-amendment ASD control (ASD-NA). Treatments were applied to soil artificially infested with *F. oxysporum* f. sp. *fragariae*. The trial employed six biological replicates per treatment arranged in a randomized complete block design. Incubation temperature, duration, and the photoperiod were the same as noted above for ‘low rate trial 1′. Soil density of *F. oxysporum* was determined at day 0, day 28 (1 week after completion of ASD), and plant harvest.

### 2.6. Plant Bioassays

At the completion of the ASD incubation period, treated soils were allowed to aerate for 2 weeks prior to planting one strawberry transplant per pot (cv. Albion; Lassen Canyon Nursery, Redding, CA, USA). In ASD incubation temperature and duration experiments, pots were arranged in a completely randomized block design on a greenhouse bench. Plants were watered with 100 mL every other day, and fertilization was conducted by applying 20 mL of Hoagland’s complete nutrient solution [34] to each plant once a month. Plants were grown at 22 °C with ambient light supplemented by LED lights with a 12 h photoperiod for 5 months. Photosynthetically active radiation (PAR, 400–700 nm) at the plant canopy level was between 100 and 250 µE m^−1^ s^−2^.

For the ASD carbon source input type experiments, treated soils were aerated, planted with the same strawberry cultivar, irrigated, and fertilized, as noted above. Pots were arranged according to a completely randomized design in environmental growth chambers. Trials concerning *M. phaseolina* were incubated for 2 weeks at 24/18 °C post-planting, followed by 30/18 °C with a 12 h photoperiod for 7 weeks. Experiments involving *F. oxysporum* f. sp. *fragariae* infested soils were incubated at 24/18 °C for 10 days post-planting followed by 28/20 °C with 12 h photoperiod for 5 weeks to encourage host infection by the pathogen and the development of wilt symptoms. Photosynthetically active radiation at the plant canopy level was performed, as noted above.

### 2.7. Assessment of Crown Infection

DNA was extracted from two duplicate 0.5 g crown tissue samples of each plant using the Power Plant Pro DNA isolation kit (MO BIO Laboratories Inc., Carlsbad, CA, USA). Strawberry crown infection was determined by qPCR for *M. phaseolina* using previously described methods [30]. *F. oxysporum* f. sp. *fragariae* infection was assessed by qPCR using the specific primer pair FofraF (5′-CAG ACT GGG GTG CTT AAA GTT-3′)/FofraR (5′-AAC CGC TAG GGT CGT AAC AAA-3′) [35]. Plants were also processed for pathogen detection and isolation from plant tissue using the protocol modified from [36]. Surface disinfested crown tissue was finely cross-sectioned with a sterile razor blade, and tissues were used for DNA extraction, and fungal isolation on 1/10th-strength PDA amended with rifampicin (100 μg mL^−1^, GoldBio, St Louis, MO, USA) and kanamycin (75 µg mL^−1^). Plates were incubated at room temperature, and crown tissue was examined daily for the emergence of hyphal growth using a light microscope (100×). *F. oxysporum* f. sp. *fragariae* infection was assessed by qPCR or the crown plating method. Morphological features including *M. phaseolina* sclerotia and *F. oxysporum* f. sp. *fragariae* conidia, developing from fungal growth emanating from crowns plated on culture media, were used to document the recovery of the pathogen.

### 2.8. Effect of ASD Treatment Variables on the Soil Metabolome and Microbiome

#### 2.8.1. Effect of Soil Temperature on Volatile Profile

Volatile profiles were assessed in the initial experiment assessing the effect of incubation temperature on the efficacy of ASD-RB for control of *F. oxysporum* f. sp. *fragariae*. Volatiles produced during ASD were analyzed after 3 weeks. Soil (400 g sample) was collected from two replicate pots of each treatment and placed individually in sealable 3.79 L glass jars. Samples were equilibrated for 30 min by passing purified air through a silanized glass trap (9 cm-length × 4 cm-diameter) filled with Tenax^®^ TA (60–80 mesh). A headspace air sample of 25 mL from ASD treatments and 300 mL from control treatments was collected on a trap consisting of a silanized glass tube (11.5 cm × 6 mm o.d.) loosely packed with Tenax^®^ TA porous polymer (60–80 mesh).

Volatile analysis was conducted using a Hewlett–Packard 5890A Gas Chromatograph (GC) equipped with a DB–WAX (Agilent Technologies, Santa Clara, CA, USA; 30 m length × 0.25 mm i.d., 0.25 μm-thick film) attached to a Hewlett–Packard 5971A mass selective detector. Samples were introduced into the GC with a Gerstel Thermal Desorption System Autosampler. Run parameters were initial temperature 50 °C; delay time 1 min; initial time 1 min; ramp 1 rate 60 °C min^−1^, end temperature 200 °C, hold time 10 min; ramp 2 rate 30 °C min^−1^, end temperature 300 °C and, hold time 10 min. Samples were desorbed using splitless desorption mode. Cooled injection system parameters were: an initial temperature at 60 °C, equilibrium time for 1 min, initial time for 0.10 min without cryo-cooling, ramp rate at 10.0 °C s^−1^, and end temperature at 240 °C, held for 1 min. Helium was used as the carrier gas, and the flow rate was maintained at 3 mL min^−1^. Column temperatures were programmed from 35 °C for 1.5 min to 220 °C at 10 °C min^−1^ ramp rate, then held at 220 °C for 1 min. The mass spectra of the unknown compounds were compared with those of known compounds using the National Institute of Standards Technology MS Search 2.0 d. The identity of compounds selected based on their potential capability of distinguishing treatments was confirmed by comparing spectra to those of pure standards. A total of 21 compounds were quantified by integrating the peak area for each treatment.

#### 2.8.2. Effect of ASD Carbon Source on Soil Metabolome and Microbiome

The soil was artificially infested with *F. oxysporum* f. sp. *fragariae* using a conidial suspension, as noted above. The study was conducted with soil contained in enclosed glass jars using the methods and conditions described in [20], and consisted of three independent experiments, each employing a single carbon source (‘low rate’ GR, RB, or WH) in addition to the ASD no-amendment control (ASD-NA). Each experiment included nine sampling time points (day 0, 1, 3, 7, 15, 21, 24, 28, and 31) with three biological replicates per treatment at each time point. The sealed jars were arranged in controlled environment chambers using a randomized complete block design and incubated at 24/18 °C day/night temperature regime with a 12 h photoperiod. Jar lids were removed after day 21 to allow for soil aeration, and a final analysis of the metabolome and microbiome was conducted on day 31.

##### Assessment of Volatile and Polar Metabolite Profiles

Analyses for headspace O_2_, CO_2_, and volatile metabolites were assessed at all time points through day 21 and the end of the ASD treatment, using methods previously described by Hewavitharana et al. [20]. Headspace O_2_ and CO_2_ concentrations were determined using a handheld gas analyzer (Dansensor A/S, Ringsted, Denmark). Volatile sample preparation and GC/MS analysis were conducted as previously described [20] using an Agilent 6890 N gas chromatograph (GC) and 5975B mass selective (MS) detector (Agilent Technologies, Palo Alto, CA, USA). Representative soil samples were collected from each jar at all nine time points, which were used to characterize polar metabolites. Soil samples were obtained using a metal spatula to collect six 1-cm-diameter cores in a star-shaped pattern. Soil cores were immersed in liquid nitrogen and stored at −80 °C. The entirety of the frozen soil sample was ground to a powder using a cryogenic freezer/mill (SPEX SamplePrep, Metuchen, NJ, USA). The ground frozen soil samples (2.0 g) were used in the analysis of polar metabolites. Trimethylsilyl (oxime) derivative analysis of polar metabolites consisted of a 50% methanol/water extraction, as described in [37], and followed by trimethylsilyl (oxime) derivatization and GC-MS analysis, as previously described (Supplementary Material on soil metabolome data, S2 [20,38]).

##### Analysis of the Soil Microbiome

A DNA extract was obtained from 0.25 g of each ground soil sample using the DNeasy PowerSoil DNA isolation kit (Qiagen, Germantown, MD, USA). Amplicon sequencing of bacterial and fungal communities was performed for each soil replicate at sampling time points 0, 1, 3, 7, 15, 21, and 31. All methods were as previously described [20], except for the primers used in the amplification of bacterial DNA. The primer pair 799F (5′-ACCMGGATTAGATACCCKG-3′)/1193R (5′-ACGTCATCCCCACCTTCC-3′) [39,40] was used to amplify the V5-V6-V7 regions of the 16S rRNA gene. Sequencing was performed at an external facility (Molecular Research, Shallowater, TX, USA) using the Illumina MiSeq platform following the manufacturer’s guidelines. Data processing was performed as previously reported [41]. PAST v.4.03 software [42] was used to conduct principal coordinate analysis of bacterial and fungal species OTU data (Supplementary Material on soil microbiome data, S3). Analysis of similarity (ANOSIM) was conducted using the same program to assess the relative similarity of bacterial and fungal communities across treatments and sampling time points.

### 2.9. Statistical Data Analysis

**Figure 1:**
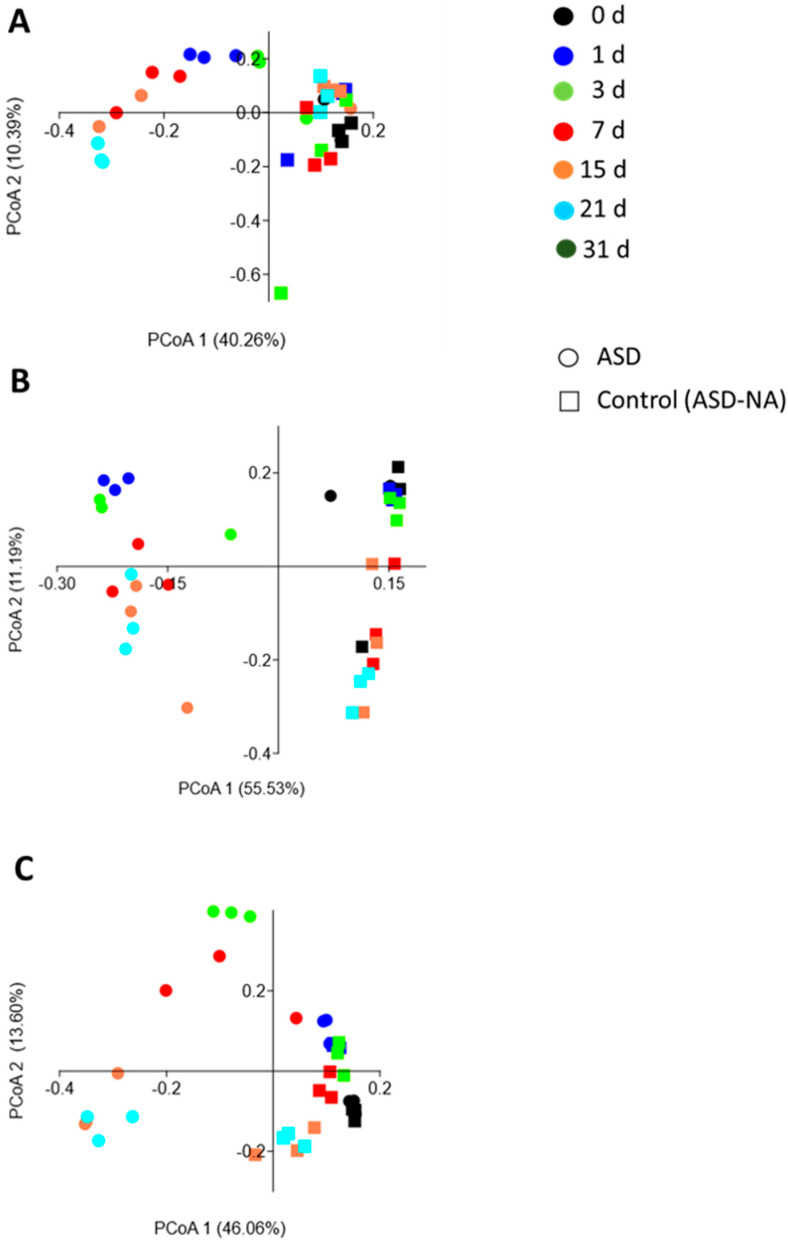
Principal coordinate scores plot representing changes in volatile metabolite profiles over a 21-d simulated anaerobic soil disinfestation (ASD) treatment period. ASD was conducted using ground grass (**A**), ground wheat leaf tissue (**B**), or rice bran (**C**) as the ASD carbon source or without the amendment (ASD-NA). Ordination of soil metabolomes was conducted by principal coordinates analysis of metabolite abundance data using the Bray-Curtis similarity coefficient.

**Figure 2:**
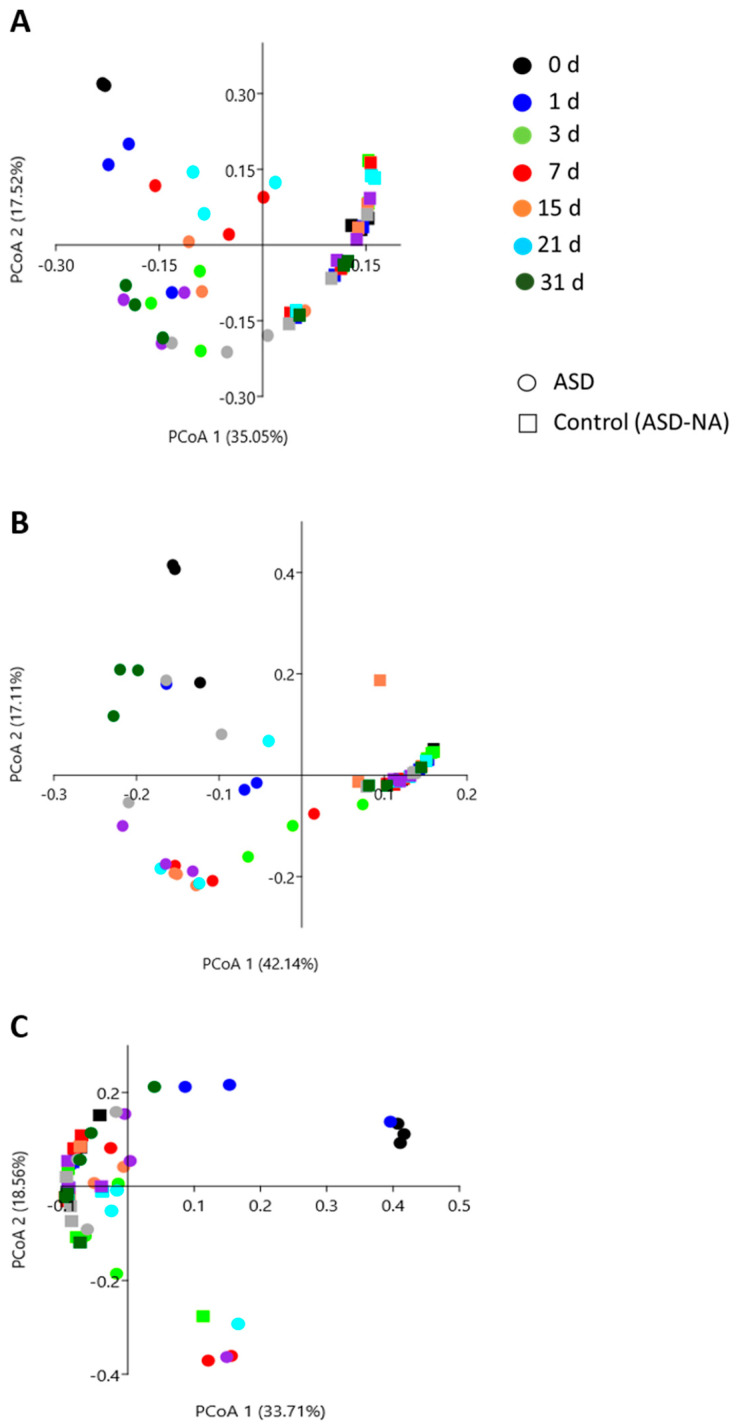
Principal coordinate scores plot representing changes in polar metabolite profiles over a 21-day simulated anaerobic soil disinfestation (ASD) treatment followed by a 10-d aeration period. The polar metabolite profile exhibited dynamic changes in composition over time in all ASD-carbon source treated soils but was generally static over time in the ASD no carbon amendment (ASD-NA) control soil. Analysis was conducted using the Bray–Curtis similarity coefficient. ASD was conducted using ground grass (**A**), ground wheat leaf tissue (**B**), or rice bran (**C**) as the ASD-carbon source.

SAS 9.4 software was used for statistical data analysis. Quantitative data such as plant fresh biomass were analyzed by three-way ANOVA using Proc Mixed procedure, and appropriate data transformations were performed to meet assumptions of the model. When data transformation did not yield homogeneous variance or normal distribution, Friedman’s nonparametric method was used to analyze the data. Count data of colony-forming units were standardized to Poisson distribution and analyzed using the Proc Genmod procedure. When there were excess zeros in count data, zero-inflated Poisson distribution or zero-inflated negative binomial distribution was used to model the data [43]. Crown infection percentage data were analyzed using multiple logistic procedures. First, the factors’ significant effects were identified using the forward selection process in modeling, and then selected factors were refit in the selected model using reference parametrization. Odds ratio estimates were used to make comparisons between treatments or treatment combinations. Odds ratio is considered to be a measure of association between an exposure and an outcome. It represents the odds that a particular outcome will occur given a specific exposure compared to the odds of the outcome occurring in the absence of that exposure [44]. Metaboanalyst software was used for volatile metabolite data analysis [45]. In data analysis, experimental design, ‘two-factor independent samples’ and data scaling method, and ‘auto’ were employed. Euclidean distance was estimated to assess the relative similarity of volatile patterns, and Ward algorithm was used to generate heatmap visualization. Microbial populations, volatile and polar metabolite data were analyzed with PAST statistical software [42] using principal coordinate analysis (PCoA; Figure 1 and Figure 2) and analysis of similarity (ANOSIM; Table 2). PCoA was conducted using the Bray–Curtis similarity coefficient.

## 3. Results

### 3.1. The Carbon Source-Mediated Changes in the Soil Microbiome and Metabolome

Headspace O_2_ concentration for all ASD treatments conducted with a ‘low rate’ carbon input decreased below that of the ASD no-amendment control within 24 h of treatment initiation (*p* < 0.05) and was near 0% for all ASD treatments by day 3. The no-amendment control showed no significant change in headspace O_2_ concentration throughout the incubation period, ranging from 16–19% through the 21-day period. Correspondingly, headspace CO_2_ concentration increased for ASD-GR, ASD-RB, and ASD-WH treatments ranging between 25 and 33% at day 21 and remaining below 5% at all sampling points for the ASD-NA control.

All ASD treatments resulted in dynamic shifts in the soil metabolome, including volatile and polar metabolites, relative to the no-amendment control. For ASD with carbon source treatments, shifts in the metabolome continued over the duration of the incubation period. Volatile metabolite profiles generated in ASD-treated soils differed significantly (*p* = 0.0001) from ASD-NA by both day and treatment based on ANOSIM (Table 2). The volatile metabolite profile exhibited dynamic changes in composition over time in all ASD-carbon source treated soils with a similar trajectory over time for all carbon source inputs (Figure 1). PCoA of volatile metabolite data indicated increasing divergence in ASD plus carbon input soils from the ASD-NA soils over time, with volatile profiles remaining relatively static in ASD-NA soils. A greater dissimilarity was observed between treatment than a day. Among significant volatile metabolites possessing known antimicrobial activity, dimethyl disulfide (DMDS) was first detected at a significantly (*p* < 0.05) higher quantity in the ASD-WH, ASD-GR and ASD-RB treatments at day 3, 7, and 15, respectively, relative to the control. Levels of DMDS in ASD treatments remained elevated relative to the control at all subsequent sampling points. Butanoic acid was significantly (*p* < 0.05) higher in the ASD-GR and ASD-RB treatments by day 7 relative to ASD-NA, but significant elevation of this metabolite in the ASD-WH treatment was not observed until day 21. Acetic acid was significantly (*p* < 0.05) elevated in ASD-RB and ASD-GR relative to the control by day 7 and ASD-WH by day 15. Pentanoic acid (valeric acid) was significantly greater than the control in ASD-RB (*p* = 0.015) and ASD-WH (*p* = 0.040) treatments by day 15 and by day 21 in ASD-GR (*p* < 0.001). Production of p-Cresol, a phenolic compound shown to have activity toward a wide range of micro-organisms [46], was significantly elevated relative to the control in ASD-WH at day 7 (*p* = 0.017), day 15 in ASD-RB (*p* < 0.001), and day 21 in ASD-GR (*p* = 0.002) treated soils. Notably, production of 2,4 hexadiene was observed in carbon-amended soils on days 1–21 (ASD-GR), 1–15 (ASD-WH), and 3–7 (ASD-RB), but not in ASD-NA control soils.

Polar metabolite profiles generated in ASD-treated soils (Figure 2) differed significantly (*p* = 0.0001) from the no-amendment ASD control by both day and treatment based on ANOSIM (Table 2). Dissimilarity in polar metabolite profiles between the ASD treatments and the control was immediately apparent after treatment application on days 0 and 1 (R_ANOSIM_ > 0.9259); this was initially characterized by the detection of readily consumed carbohydrates such as d-glucose and sucrose in the ASD-amended soils which were not detected in the control soil. Substrate sugars were rapidly consumed during the first days of ASD and were associated with abundant lactic acid and pyruvic acid production in all carbon-amended ASD soils. A more rapid glucose consumption observed in ASD-RB treatment, relative to the ASD-GR and ASD-WH treatments, was associated with a reversion of the polar metabolic profile to one highly similar to that of the control soil (R_ANOSIM_ = 0.0370) by day 3 (Table 2). By contrast, the polar metabolite profile in the ASD-GR and ASD-WH treatments continued to remain distinct from the ASD-NA control through day 31, with R_ANOSIM_ values approaching 1 for all comparisons at the 24-day through 31-day sampling points. By comparison, R_ANOSIM_ values for pair wise comparison of ASD-RB and ASD-NA treatment were lower ranging from 0.1111 to 0.6667 at the same sampling time points. The readily available sugars d-glucose and/or sucrose were identified in the metabolome from ASD-GR, and ASD-WH treated soils but not the ASD-RB and ASD-NA soils during soil aeration at days 28 and 31.

**Figure 3:**
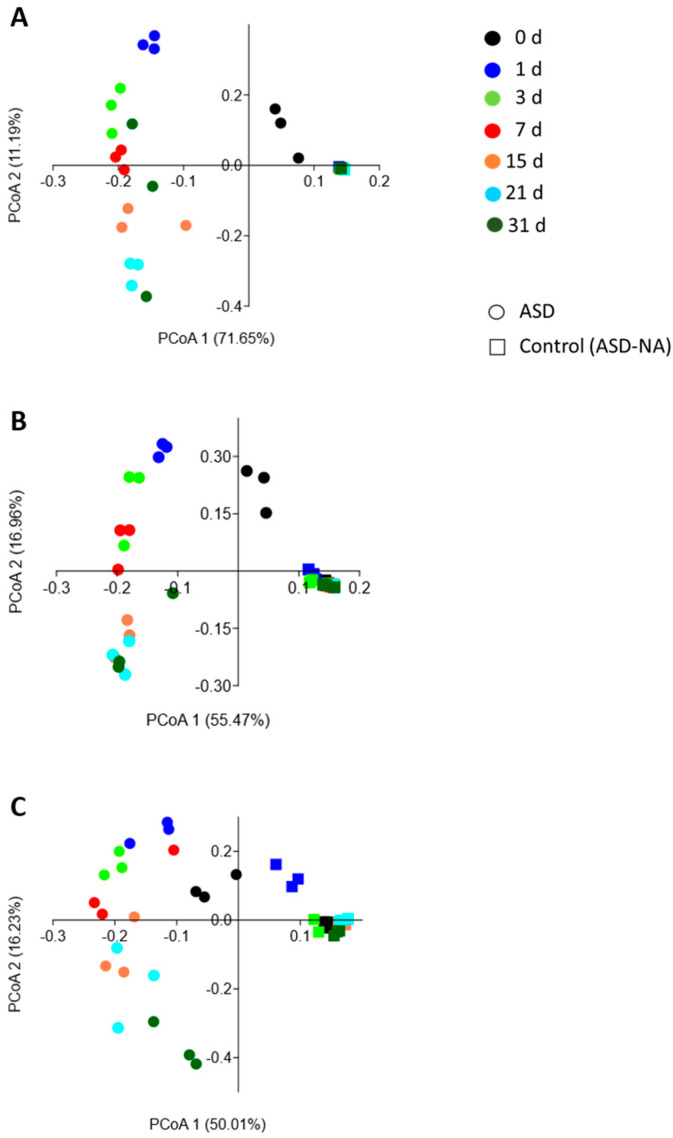
Principal coordinate scores plot representing changes in bacterial community composition over a 21-day simulated anaerobic soil disinfestation (ASD) treatment followed by a 10-d aeration period. The bacterial community exhibited dynamic changes in composition over time in all ASD-carbon source treated soils but was generally static over time in the ASD no carbon amendment (ASD-NA) control soil. ASD was conducted using ground grass (**A**), ground wheat leaf tissue (**B**), or rice bran (**C**) as the ASD carbon source.

Principal coordinate analyses of bacterial OTUs indicated dynamic changes in carbon-amended soils (ASD-GR; ASD-RB; ASD-WH) over time while the same communities in the no-amendment control (ASD-NA) remained static. The first principal coordinate of PCoA corresponds to the time series (day), and the second principal component corresponds to treatment (amendment vs. no-amendment) (Figure 3). The dynamic changes in bacterial community composition followed a similar trajectory over time in all three ASD carbon input soil treatments. R_ANOSIM_ values between ASD treatments and the corresponding ASD-NA control approached or equaled 1 for all time points, indicating a great degree of dissimilarity with no overlap in bacterial community composition.

**Figure 4:**
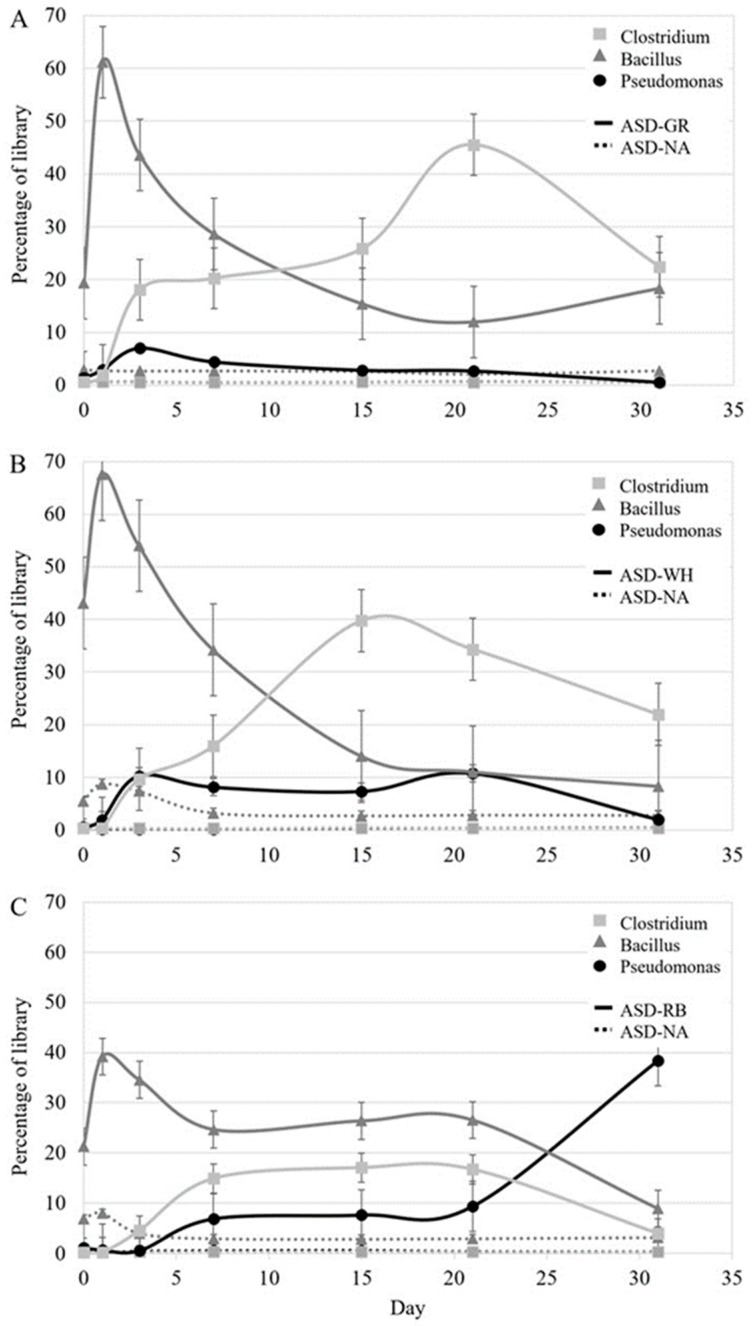
Changes in relative abundance of select bacterial OTUs during simulated anaerobic soil disinfestation (ASD) treatment using (**A**) ground grass (ASD-GR; 10 t h^−1^), (**B**) wheat leaf tissue (ASD-WH; 10 t h^−1^), and (**C**) rice bran (ASD-RB; 5.0 t h^−1^) as the carbon source compared to ASD without an amendment (ASD-NA). Values represent mean abundance of OTUs across all replications with *n* = 3. Error bars represent standard error of the mean.

Relative abundance of *Bacillus* spp. increased rapidly and significantly (*p* < 0.05) by day 1 in all carbon source ASD treatments compared with the no-amendment ASD control (Figure 4). This was followed by a precipitous decline in the relative abundance of *Bacillus* spp. on days 3 and 7 and corresponded with the rapid increase in the proportion of *Clostridium* spp. in the total bacterial community. *Clostridium* spp. represented a significantly greater proportion of the bacterial community in ASD-GR soil at day 7 and by day 15 in ASD-WH and ASD-RB soil relative to the ASD-NA control. The relative proportion of *Kaistobacter* spp., which comprised 10–20% of the bacterial community in ASD-NA, declined significantly in all three ASD-treated soils relative to ASD-NA, representing 1–2% of the bacterial community in ASD carbon input soils by day 3. Upon soil aeration, the proportion of *Kaistobacter* spp. in ASD-RB soil recovered and represented 8% of the bacterial community at day 31. Relative abundance of *Pseudomonas* spp. in the bacterial community was slightly elevated during the anaerobic phase in ASD-treated soil compared to ASD-NA. However, post-aeration, the relative abundance of *Pseudomonas* spp. in the ASD-RB soil increased rapidly and was significantly higher than the control at day 31. Relative abundance of *Pantoea* spp. increased from <1% at day 0 to approximately 5% in ASD-GR and ASD-WH soils and remained elevated over the 31-day trial. No change in relative abundance (<1%) of *Pantoea* spp. was observed over the study period in the ASD-RB or the ASD-NA control soil.

Both day and treatment had a significant (*p* = 0.0001) effect on fungal community composition, but R values for day indicate greater dissimilarity due to treatment (Table 2). As indicated by PCoA of fungal OTUs, fungal communities changed slightly during the anaerobic phase of ASD (Figure S1). Dissimilarity in fungal community composition between the ASD-carbon source treatments and the no-amendment control was largely driven by changes in the proportion of *Fusarium* spp. in response to the carbon amendment. The relative abundance of *Fusarium* OTUs was significantly higher (*p* < 0.05) in ASD-GR, ASD-RB, and ASD-WH soils than ASD-NA soil by day 3. After a peak at day 7, the relative proportion of *Fusarium* spp. in the fungal community declined in ASD-GR and ASD-WH soils through day 21 and corresponded with proportional increases in *Mortierella* spp. and *Botryosphaeria* spp. in all carbon-amended soils. Upon soil aeration at day 21, a subsequent increase in the relative abundance of *Fusarium* spp. was observed in ASD-GR and ASD-WH, but not ASD-RB soils, at day 31. ASD-RB treatment suppressed the relative abundance of the postharvest pathogens *Penicillium* spp. and *Colletotrichum* spp. compared with the no-amendment controls beginning on day 3 of ASD and into the aeration phase.

In the metabolome/microbiome experiment, *F. oxysporum* soil density was several orders of magnitude higher in ASD-GR, ASD-RB, and ASD-WH soils when compared to ASD-NA on day 3. The concentration of *F. oxysporum* DNA (pg g^−1^ soil) detected in soils at day 0 was approximately 10^2^ pg g^−1^ soil but increased rapidly to 10^3^ pg g^−1^ soil in ASD-RB soil at day 1 and nearly 10^4^ pg g^−1^ in ASD-GR and ASD-WH soils by day 3 and day 7, respectively. Soil density of *F. oxysporum* declined in all ASD-carbon source treatments through the anaerobic phase of the trial and was not significantly different from the control treatment at day 21. Although the relative abundance of *F. oxysporum* OTUs was elevated in ASD-GR and ASD-WH treatments at the final sampling, the *F. oxysporum* density determined by qPCR did not differ among all soil treatments, ranging between 10^1^ and 10^2^ pg g^−1^ soil, at this timepoint (day 31).

### 3.2. The Effect of Carbon Source on ASD-Mediated Disease Suppression

#### 3.2.1. Macrophomina Crown Rot

Infection of strawberry crown tissue by *M. phaseolina* was significantly influenced by soil treatment (*p* = 0.0019) in trial 1 based on direct isolation of the pathogen (Table 3). When the NTC was used as the reference, ASD-WH (*p* = 0.0272) had a significantly lower incidence of crown infection, but ASD-GR (*p* = 0.2912) and PC treatment (*p* = 0.8369) were not significantly different from NTC. In trial 2, the percentage of *M. phaseolina* crown infection in both ASD-WH and PC treatments was 0% (Table 3) which caused a quasi-complete separation of data in the analysis. Therefore, the PC treatment was removed from the analysis. When NTC was used as the reference treatment, crown infection was significantly elevated in ASD-RB (*p* = 0.0076) and was significantly reduced in the ASD-WH (*p* < 0.0001) treatment. There was no significant difference in percent crown infection between the NTC and ASD-GR treatments (*p* = 0.1488). 

In trial 1, there was no significant (*p* = 0.4467) difference among soil treatments in the quantity of *M. phaseolina* DNA detected in crown tissue. A significant treatment effect was observed in trial 2 (*p* = 0.0004), where the quantity of *M. phaseolina* DNA detected in crowns was significantly higher in the ASD-RB and NTC treatments relative to that in the ASD-GR, ASD-WH, and PC treatments. The quantity of *M. phaseolina* DNA detected in crowns from the ASD-RB, and NTC treatment did not differ significantly (Table 3). Soil treatment did not have a significant effect on strawberry growth in trial 1 (*p* = 0.5946). In trial 2, total fresh biomass (*p* = 0.0118) and total fresh root biomass (*p* = 0.0008), but not fresh shoot biomass (*p* = 0.0834), were significantly higher in the PC treatment relative to all other soil treatments (Figure S2). ASD treatments did not significantly affect plant growth parameters in trial 2.

**Figure 5:**
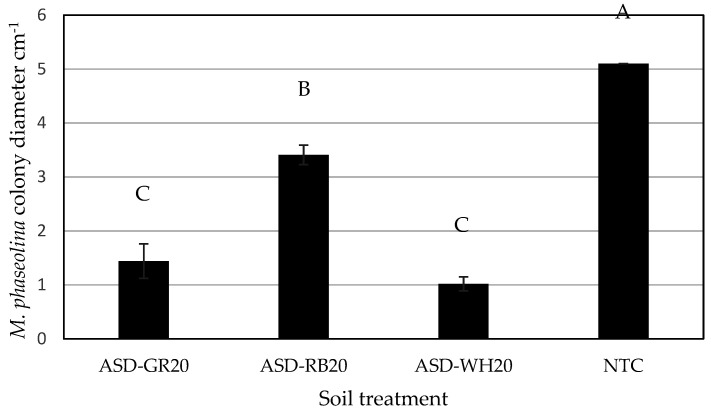
The activity of volatiles produced during anaerobic soil disinfestation (ASD) was conducted using different carbon inputs on colony growth of *Macrophomina phaseolina*. Soil treatments included ASD with carbon input of grass residues at 20 t ha^−1^ (ASD-GR), rice bran at 20 t ha^−1^ (ASD-RB), wheat residues at 20 t ha^−1^ (ASD-WH), and no-treatment control (NTC). Friedman’s nonparametric method was used for data analysis. Values represent mean pathogen colony diameter across all replicates with *n* = 10. Error bars represent standard error of the mean. Means designated with the same letter are not significantly different (*p* < 0.05).

When exposed to volatiles produced during ASD, the mycelial growth of *M. phaseolina* was significantly affected by soil treatment (*p* < 0.0001). Colony diameter of the fungus at the end of the incubation period varied as NTC > ASD-RB > ASD-GR and ASD-WH (Figure 5). The latter two treatments were not significantly different with respect to mycelial growth. Conversely, after transferring the fungal cultures onto fresh media post-exposure to ASD volatiles, there was no significant effect of soil treatment on the growth of *M. phaseolina* (*p* = 0.9100). However, fungal cultures exposed to volatiles generated during any ASD treatments failed to produce microsclerotia, whereas abundant microsclerotia production by *M. phaseolina* was observed in the NTC treatment.

#### 3.2.2. Fusarium Wilt

In ASD carbon input ‘low rate trial 1′, average initial soil density of *F. oxysporum* f. sp. *fragariae* was 3533 CFU g^−1^ soil. After 21 days, the soil population of the pathogen was significantly reduced in both ASD treatments: RB and GR having 0 CFU g^−1^ soil compared with NTC having 5667 CFU g^−1^ soil. The infection of strawberry crown tissue by *F. oxysporum* f. sp. *fragariae* was significantly (*p* = 0.002) influenced by soil treatment. Both ASD-GR (10%) and ASD-RB (16%) treatments significantly reduced strawberry crown infection relative to the NTC (85%), and there was no significant (*p* = 0.888) difference in crown infection between the two ASD treatments. Correspondingly, plants grown in ASD-treated soil tended toward greater fresh weight biomass at harvest (ASD-GR = 17.15 g; ASD-RB 20.12 g) relative to the NTC (13.79 g); however, only ASD-RB differed significantly (*p* = 0.006) from the control. There was no significant difference in plant biomass (*p* = 0.163) between the ASD-GR and ASD-RB treatments.

In ASD carbon input ‘low rate trial 2′, ASD did not yield significant benefits in limiting amplification of *F. oxysporum* soil populations or resulting in disease control. *F. oxysporum* soil density initially trended upward in ASD soils, receiving a carbon input relative to the ASD-NA treatment. However, there were no significant (*p* > 0.05) differences in the soil density of the pathogen among treatments on day 0, day 28 or at plant harvest. ASD carbon input did not have a significant effect on the concentration of *F. oxysporum* f. sp. *fragariae* (*p* = 0.2806) detected in strawberry crown tissue at harvest. Disease severity and total fresh weight of strawberry plants grown in ASD-RB, ASD-GR, and ASD-WH soils did not differ significantly (*p* > 0.05) from the ASD no-amendment control (ASD-NA) at harvest.

### 3.3. The Effect of Temperature and Incubation Duration on ASD-RB-Mediated Disease Suppression

#### 3.3.1. Variation in Volatile Profiles Produced

**Figure 6:**
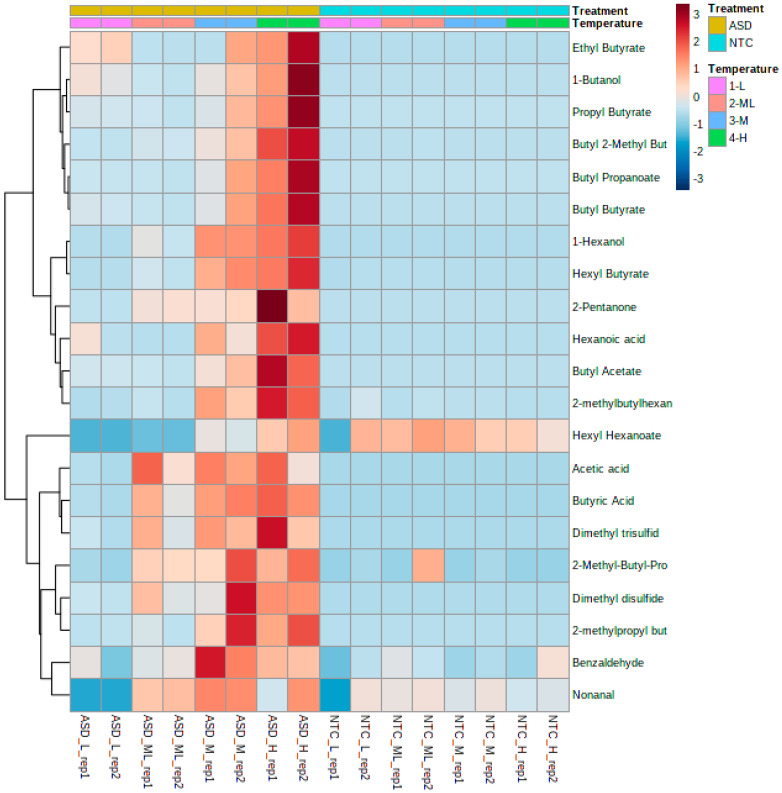
Effect of soil treatment and incubation temperature on the post-treatment volatile profiles of selected compounds in ASD-treated and no-treatment control soil. Soil treatments were anaerobic soil disinfestation conducted using rice bran as the carbon input (20 t ha^−1^) (ASD) and no-treatment control (NTC); day/night incubation temperatures were low (L), medium-low (ML), medium (M), and high (H). L = Low incubation temperature (16/10 °C); ML = Medium-low incubation temperature (24/18 °C); M = Medium incubation temperature (32/26 °C); H = High incubation temperature (40/34 °C). Butyl 2-Methyl But = Butyl-2-methyl-butyrate; 2-methylbutylhexan = 2-methylbutylhexanoate; Dimethyl trisulfid = Dimethyl trisulfide; 2-methylpropyl but = 2-methylpropylbutyrate; 2-Methyl-Butyl-Pro = 2-methylbutylpropanoate. Euclidean distance was estimated to assess the relative similarity of volatile patterns, and Ward algorithm was used to generate heatmap visualization. The color scale 3 (dark brown) to −3 (dark blue) indicates the highest to lowest concentration of the volatiles, respectively.

Several distinct volatiles were produced in response to the ASD-RB treatment, with the metabolic profile varying considerably across the ASD incubation temperatures examined in this study (Figure 6). A variety of alcohols, ketones, aldehydes, acids, sulfides, and esters were unique to the ASD-RB treatment, relative to the control, with composition and abundance varying in a temperature-dependent manner. Identification of 1-hexanol, 2-methyl-butyl-hexanoate, and hexyl butyrate were only accomplished in the ASD-M and ASD-H temperature treatments. Acetic acid, butyric acid, dimethyl disulfide, and dimethyl trisulfide were detected at all ASD incubation temperatures, except ASD-L, with a higher abundance of these volatiles detected in ASD-H temperature treatment. Alcohol compounds (e.g., 1-hexanol) and esters (e.g., ethyl butyrate, propyl butyrate, butyl butyrate) were more abundant in the ASD-M and ASD-H treatments than under ASD L or ML temperature conditions (Figure 6).

#### 3.3.2. Effect of ASD Incubation Temperature and Duration on Changes in Soil Physiochemical Parameters

Soil incubation temperature influenced the cumulative anaerobicity attained in response to the ASD treatment conducted with rice bran at an input rate of 20 t ha^−1^ (data not shown). In general, at a higher soil incubation temperature, a greater cumulative anaerobicity was attained over the ASD incubation period. For instance, the average cumulative redox potential of 73,000 mV h, 137,000 mV h, 260,000 mV h, and 376,000 mV h was generated at the end of the 6-week incubation period in ASD-L, ASD-ML, ASD-M, and ASD-H treatments, respectively.

#### 3.3.3. Effect of ASD Incubation Temperature and Duration on Pathogen Density in Soil and Crown Tissue

*M. phaseolina* soil density in response to ASD-RB treatment was not significantly affected by incubation temperature or duration in either trial as determined by qPCR. However, strawberry crown infection by *M. phaseolina* was influenced by certain factors in both trials (Figure S3A). The *M. phaseolina* crown infection in trial 1 was significantly affected by temperature (*p* = 0.0028) and incubation duration (*p* = 0.0005); there was a significant three-way interaction (*p* < 0.0001) of soil treatment, temperature, and incubation duration, and a two-way interaction between treatment and duration. Crown infection at L temperature was greater for 3-week incubation (20.0% ± 12) than 6 weeks incubation (1.0% ± 1) irrespective of soil treatment with ASD-RB or NTC. Crown infection was greater at L (14.0% ± 0.1) temperature than M (5.0% ± 0.1) and ML (4.0% ± 0.0) temperatures when ASD-RB treatment was conducted for 3 weeks. In trial 2, only two-way interactions between treatment-duration (*p* < 0.0001) and temperature (*p* = 0.0044) were significant (Figure S3A). ASD-RB treatment resulted in lower *M. phaseolina* crown infection (5.5% ± 5.5) than the NTC at 21.3% ± 5.7 when incubated for 3 weeks. In trial 1, none of the test factors significantly affected the quantity of *M. phaseolina* DNA detected in strawberry crown tissue. However, in trial 2, the quantity of *M. phaseolina* DNA detected in crown tissue was influenced by soil treatment (*p* = 0.0002), with the quantity of the pathogen significantly lower for ASD-RB relative to NTC.

In both trials, soil treatment significantly affected the density of *F. oxysporum* f. sp. *fragariae* (trial 1: *p* = 0.0457, trial 2: *p* < 0.0001) with the ASD-RB treatment significantly reducing the pathogen soil inoculum. Incubation temperature, but not the duration of the incubation period, affected *F. oxysporum* f. sp. *fragariae* population density where the population density was the lowest in the H temperature profile (*p* < 0.0001). There was a significant two-way interaction between temperature and treatment in trial 2. ASD-RB at H temperature resulted in a decreased soil density of *F. oxysporum* f. sp. *fragariae* (0 CFU g^−1^ soil; *p* < 0.0001), whereas ASD-RB at L temperature had an increased density of the fungus (21,500 CFU g^−1^ soil; *p* < 0.0001). For ASD conducted at L, M, and H incubation temperatures, *F. oxysporum* f. sp. *fragariae* density was reduced in soil compared with NTC, but not at ML temperature in trial 1. ASD-RB conducted using the ML, M, and H, but not L temperature profiles, significantly reduced the density of *F. oxysporum* f. sp. *fragariae* in soil compared with the NTC in trial 2.

In trial 1, soil treatment (*p* < 0.0001), incubation temperature (*p* < 0.0001), and three-way (*p* < 0.0001) and all two-way interactions (*p* < 0.0001) of soil treatment, temperature, and duration significantly influenced *F. oxysporum* f. sp. *fragariae* crown infection (Figure S3B). When ASD-RB was performed at L incubation temperature for 6 weeks, crown infection was greater than the NTC incubated under the same conditions. ASD-RB treatment at L temperature also had greater crown infection than ASD-RB treatment at M and ML temperatures for 6-week incubation. In NTC, crown infection was higher when incubation was conducted for 3 weeks at M temperature compared to ML temperature. Following a similar pattern to the first trial, *F. oxysporum* f. sp. *fragariae* crown infection was significantly influenced by soil treatment (*p* < 0.0001), incubation temperature (*p* = 0.0002), and the two-way interactions (*p* = 0.0002), but not by incubation duration in trial 2. ASD-RB significantly reduced crown infection at all incubation temperatures compared to the corresponding no-treatment control (Figure S3B). ASD conducted at H temperature had a lower incidence of infection relative to ASD treatments conducted at all other temperatures, whereas ASD-RB conducted at L incubation temperature favored pathogen infection relative to M and ML ASD-RB temperature treatments.

#### 3.3.4. Influence of ASD Temperature and Duration on Strawberry Plant Growth

Fresh biomass of strawberry grown in *F. oxysporum* f. sp. *fragariae* infested soil and *M. phaseolina* infested soil was significantly greater in ASD-RB 20 treated soils than the NTC in both trials (*p* < 0.0001 and *p* = 0.01; Table S1). In the Fusarium wilt trial, fresh plant biomass in the ASD treatment was twice as much as the NTC treatment. In both trials of these two pathogen-infested soils, the impact of soil treatment was consistent. There was no significant effect of temperature or incubation period on plant growth in these soils.

## 4. Discussion

Anaerobic soil disinfestation has been utilized effectively to control certain soil-borne diseases of strawberry [16,47,48] but has lacked consistent effectiveness for controlling other diseases, including Fusarium wilt, when applied in specific regions or under certain conditions [22]. Several tactics have been examined in an attempt to improve ASD for control of soil-borne pathogens, including modulation of carbon source, soil temperature, and cumulative hours of anaerobicity. Studies regarding the mechanisms of ASD-mediated disease suppression have highlighted the importance of potential biochemical modes of action, such as the proliferation of select microbes (e.g., anaerobic Firmicutes) that produce carbon source-dependent volatiles with antifungal activity [19,21,49]. The study in [20] linked changes in the soil microbiome with corresponding changes in the soil metabolome in response to ASD-rice bran treatment, with the subsequent characterization of distinct physiological stages in the ASD process. As carbon source influences the efficacy of ASD-induced disease control [24], an understanding of the physiological and biological responses induced by ASD using different carbon sources could facilitate optimization of treatments for controlling specific pathogens.

Under certain environmental conditions, the proliferation of *F. oxysporum* soil density has been observed in this and previous studies in response to the addition of a carbon amendment in ASD-treated soils [24,33]. Thus, the application of ASD to control this pathogen will require a balance between the preference of *F. oxysporum* for a specific amendment and the necessity of a carbon input for generating anaerobic conditions during ASD. Likewise, dynamic changes in the soil microbiome and metabolome essential for effective disease control rely upon the ASD carbon input [20,21,49]. Rice bran, orchard grass, and wheatgrass are rich in polymers (e.g., polysaccharides, proteins) and monomers (e.g., monosaccharides, amino acids), establishing them as ideal substrates for soil micro-organisms [50,51]. Fungi, including *F. oxysporum* [52,53], utilize d-glucose and sucrose as a substrate, which was readily available and rapidly consumed in all ‘low rate’ carbon input ASD treatments examined in this study. Depletion of sugars in ASD-RB soils was observed after day 1, whereas sugars in orchard grass and wheatgrass were metabolized over the course of ASD and into the aeration phase. Previously, glucose consumption in soils treated with ASD-RB at a rate of 20 t h^−1^ had diminished by day 3 of ASD [20], suggesting that rice bran is readily digested by soil microbes. The slower consumption of grass substrates other than rice bran may result from differences in cellulose composition [54,55]. The extended availability of these substrates in the ASD-GR- and ASD-WH-treated soils may be responsible for the continued dominance of *F. oxysporum* within the fungal community observed in this trial relative to the ASD-RB treatment.

Carbon input rate is an important factor for reducing the cost of ASD treatments, and hence adoption [15]. Our ‘low rate’ amendment trials show that a reduction in the input rate may also further disease suppressiveness by preventing the saprophytic proliferation of pathogens upon aeration of ASD-treated soils. It has been reported that both pathogenic and nonpathogenic forms of *F. oxysporum* on strawberries are competitive saprophytes [21,56]. The observed overall efficacy of ‘low rate’ carbon input ASD treatments, irrespective of the carbon source in reducing *F. oxysporum* f. sp. *fragariae* populations in ASD treatments, can be linked to limited substrate availability at the end of the anaerobic period. Further field-scale studies are needed to confirm these outcomes as it is a fine balance between not having the sufficient labile substrate required to create anaerobic conditions and a state of excess substrate, which induces pathogen saprophytic activity.

In the ‘low rate’ carbon input ASD trial conducted in sealed glass jars, dynamic shifts in the microbiome composition were observed in carbon-amended ASD treatments. Firmicutes represented the majority of soil bacterial communities through the ASD treatment period. However, *Bacillus* spp., which initially represented the dominant component of the Firmicutes community, was displaced as ASD progressed by anaerobic Clostridia, namely *Clostridium intestinale* and *Clostridium roseum*. Similar trends in ASD-induced changes to the composition of Firmicute populations over the incubation period were initially reported by [20] and have subsequently been documented in additional studies on ASD [57]. An additional, notable shift in microbiome composition was observed during soil aeration between days 21 and 31. The aeration period for ASD-GR and ASD-WH soils was characterized by the resumed aerobic breakdown of the carbon source that corresponded with increased lactic acid production by lactic acid bacteria observed in ASD at day 1. Other notable elevated populations in ASD-GR and ASD-WH soils during this period included *Enterobacter hormaechei* and *Enterobacter ludwigii,* species that participate in lactic acid fermentation [58]. Unique populations of *Massilia timonae*, a chitinase-producing bacterium [59], were observed at day 31 in ASD-RB soils when compared to ASD-NA.

A previous study identified distinct volatile spectra generated in response to ASD treatment utilizing diverse carbon sources, namely ethanol, composted steer manure, orchard grass, and rice bran [19]. In the current study, which utilized carbon input of different Graminae species, volatile or polar metabolite spectra unique to a specific carbon input were not detected, but the production of some compounds was unique compared with the no-amendment control. Notably, production of 2,4 hexadiene was observed in carbon-amended soils but not in ASD-NA soils. Generation of hydrocarbons such as 2,4 hexadiene was proposed as a potential mode of disease suppression induced by ASD-rice bran [20]. Some hydrocarbons show antagonistic properties towards fungi [60], and their production could be indirectly facilitated by the proliferation of Clostridia. In the current study, peak production of 2,4 hexadiene corresponded with increased abundance of Clostridia and declination of *F*. *oxysporum*. Likewise, increasing proportions of Clostridia coincided with the production of butanoic acid, which has demonstrated antimicrobial activity [61]. Accumulation of sulfur compounds (dimethyl disulfide, dimethyl trisulfide), p-cresol, and volatile fatty acids (acetic acid, pentanoic acid, propanoic acid, butanoic acid) observed in this and previous studies when RB was the carbon input [19,20] was also observed in ASD-GR and ASD-WH soils of the current study.

Although metabolic profiles were similar in total across the three ASD treatments, differences in production timing may be of note to managing the application of this disease control system. For instance, the production of p-cresol, a phenolic possessing broad antimicrobial activity, was observed a week earlier in ASD-WH soils than in ASD-GR soils during the incubation period. Likewise, although peak production of dimethyl disulfide (5 × 10^7^ ng mL^−1^) was similar in both soils, maximum concentration was reached on day 3 versus day 7 in the ASD-WH and ASD-GR soils, respectively. Fermenting organisms present during the anaerobic stage of ASD, including *Clostridium saccharoperbutylacetonicum* [62] and *C. aciditolerans* [63], were found in higher abundance of GR and RB amended ASD soils; these organisms may have contributed to elevated volatile fatty acid production. Generation of sulfur compounds could be attributed to *Pseudomonas* spp., *Streptomycetes* spp., and *Burkholderia* spp. [64]. All three genera were present in carbon-amended soils throughout the experiment, but it is more likely that elevated populations of *Clostridium* spp. contributed to the bulk of sulfur compound production [64,65].

Previous studies have shown the key role played by the ASD carbon source in disease suppression [22,57,66]. During the current study, evidence to strengthen this hypothesis was found in experiments with *M. phaseolina* based on the incidence of crown infection and the quantity of pathogen DNA detected in the crown. The superior performance of ASD-WH relative to ASD-RB and ASD-GR in controlling Macrophomina crown rot demonstrates the need for tailoring the carbon source according to the pathosystem in hand. While carbon source did not appear to alter ASD treatment outcomes in reducing *M. phaseolina* soil density, the lack of treatment effect could be attributed to limitations of the qPCR assay. Differences in the efficiency of DNA extraction from soil and plant tissue could be inferred by failure, at times, to detect the pathogen in soils even though a high level of crown infection was detected based on both culture plating and qPCR analysis. The observed disease suppression stemmed partly due to fungistatic bioactive volatile compounds generated in ASD-WH treatment. Although rice bran has been used commonly as a carbon input when ASD is applied in California strawberry systems [47], its effectiveness across different pathosystems, costs, and availability [67] will affect application feasibility. Effective soil-borne disease control has been attained in several systems where different grass species were used as the ASD carbon input [14,68,69]. ASD utilizing orchard grass effectively controlled apple root infection by *R. solani* AG-5 and *P. penetrans* [69]. When Italian ryegrass was used as the ASD carbon source, an increase in the growth of *Acer platanoides* L. (Norway maple) and *Catalpa bignonioides* Walt. (Southern catalpa) was observed in a field infested with *V. dahliae* [70]. The study by Muramoto et al. [16] reported ASD-RB at 20 t ha^−1^ significantly reduced *M. phaseolina* in strawberry crown rot compared with the grower standard. The observed differences in the current study could be due to differences in the strawberry cultivar used or the soil inoculum level of the pathogen. The differential effect of Graminae C-sources was not evident for *F. oxysporum* f. sp. *fragariae* at the rates of application used in this trial. Similarly, it was shown that several carbon sources could be effective for pathogens such as *V. dahliae* [23]. Hence, an analysis of potential carbon sources will be required for any given pathogen prior to the application of ASD as a disease control treatment to ensure efficacy in disease suppression.

In this study, incubation temperature was found to be a more crucial factor than the length of the incubation period in determining ASD disease control efficacy. The importance of soil temperature to the ASD process was evident in cumulative soil Eh, post-treatment pathogen survival, and plant growth. These parameters and outcomes were consistently improved when ASD was conducted at the highest incubation day/night temperature regime of 34/40 °C, and the treatment was not typically effective under the lowest incubation temperature profile. Based on a meta-analysis conducted by Shreshta et al. on *F. oxysporum* f. sp. *lycopersici,* [24], increased efficacy in disease suppression was observed when the soil temperature was above 25 °C, and more labile amendments were utilized. Similarly, ASD conducted using a diurnal temperature regime of 24/15 °C within a 3-week incubation period was ineffective in controlling *Sclerotinia sclerotiorum* and *Fusarium* spp. in the common bean when low carbon amendment rates (less than 1 mg C g^−1^ soil) were used [71]. The authors suggested that at lower soil temperatures, effective disease control in response to ASD requires carbon amendment rates as high as 4 mg C g^−1^ soil [71]. However, even at an amendment rate of 16 mg C g^−1^ soil, effective control of *F. oxysporum* f. sp. *fragariae* was not achieved in the current study when ASD was conducted using a moderate soil temperature regime (24/18 °C). This finding strongly supports the perception that higher soil temperature [72] may be required to obtain sufficient efficacy of ASD treatments and agrees with the prior study [73] concluding that ASD conducted at elevated soil temperatures (>35 °C) provides superior control of soil-borne pathogens relative to the same treatment imposed at low or moderate temperatures. Specifically for *F. oxysporum* f. sp. *fragrarie*, field studies conducted above 30 °C at 20 cm for 280–300 h have shown consistent disease suppression [74], as we have shown in controlled environment studies. This phenomenon appears to be consistent with other pathogens such as *V. dahliae*. The viability of *V. dahliae* microsclerotia was reduced when ASD was conducted at 25 °C but not 15 °C, even though strong anaerobicity was achieved at both soil temperatures [23], signifying the important role of temperature.

In the current study, reduction in disease incidence was attained when ASD was conducted at high temperatures, and no reduction in disease was observed in NTC soil at the same high incubation temperatures. Soil heating has been used extensively to control plant pathogens, insects, viruses, and weed seeds by applying various physical soil disinfestation methods including soil solarization, steaming, pasteurization, and hot water treatment [75,76]. Microsclerotia of *M. phaseolina* can withstand temperatures of 60–65 °C [77,78]. The temperatures employed in the current study represent those commonly experienced in California strawberry production and are not sufficient in and of themselves to suppress any of the pathogens targeted. In addition, exposure to elevated but non-lethal soil temperatures can induce thermotolerance in *F. oxysporum* [76], resulting in reduced efficacy of subsequent heat treatments such as those provided by soil solarization. The persistence of these pathogens in NTC strawberry field soils incubated at higher temperatures may have been caused by such induction of heat resistance. This finding indicates that suppression of these pathogens is unlikely to be attained merely by increasing soil temperature to a sublethal level and instead requires additional mechanisms activated by ASD when conducted at high incubation temperature.

The effect of incubation temperature on the efficacy of ASD may stem from two major factors directly governed by temperature. At elevated temperatures, soils subjected to ASD are more likely to become anaerobic at a faster pace than at lower temperatures due to increased microbial activity, resulting in a higher rate of oxygen depletion. Rapid attainment of anaerobicity may affect the viability of certain pathogens and propagules [79,80,81], particularly those that are strictly aerobic and result in reduced disease incidence. Elevated soil temperatures may have also influenced the spectrum and quantity of diverse antimicrobial metabolites produced. Metabolic reactions, such as those responsible for generating soil volatiles, are driven by enzymes that function at a higher rate with increasing temperature [82]. Therefore, a greater accumulation of volatiles would be expected at high temperatures. In a study that used cabbage residues as the soil amendment along with soil solarization, concentrations of soil volatiles were significantly higher when amended soils were incubated at 45 °C compared with soil incubated at room temperature [83]. In the current study, the generation of numerous potential antimicrobial volatiles was influenced by ASD incubation temperature. For example, the accumulation of acetic acid and butyric acid was elevated at high incubation temperatures compared with low incubation temperatures. Increased generation of volatiles were associated with reduced pathogen density in soil and enhanced suppression of strawberry crown infection.

In commercial crop production, the ASD incubation period is an important factor influencing the adoption of the practice, especially as growers prefer shorter incubation periods to enable expanded cropping windows. Outcomes of our study demonstrating no significant effect of incubation duration on treatment efficacy are in partial agreement with previous reports. ASD incubation periods greater than 10 weeks or having a duration of 3–5 weeks were less effective in pathogen suppression than a 3-week period [73]. There was no distinction between the 3-week and 6-week incubation periods used in this study relative to the efficacy of ASD in general. A 3-week incubation period is consistent with conventional fumigant treatment times [67]. Even so, growers must allow for soil aeration following the ASD treatment before planting the crop to avoid potential phytotoxicity.

## 5. Conclusions

The current study demonstrates that multiple components of the ASD treatment process drive changes in the soil microbiome and metabolome that will ultimately determine disease control outcomes. Specifically, a continued trajectory toward disease suppression involves the production of functional bioactive metabolites, such as DMDS, that correspond with the amplification of functional microbial elements, such as *Clostridium* spp., which transpire in a time-dependent manner in ASD-treated soils. An optimum ASD application temperature is dependent on multiple factors, including the physiology of the targeted soil-borne pathogen, soil type, and the carbon source used. In general, ASD conducted at higher soil temperatures and a shorter incubation period effectively controlled Fusarium wilt when rice bran was employed as the carbon source. Among those carbon sources examined, optimal control of charcoal rot was attained when ASD was conducted in soils amended with wheat and incubated at a moderate temperature with a shorter incubation period. Our findings demonstrate that considering these multiple factors, including the biology of the target pathogen, is imperative when formulating a prescription for the use of ASD as a disease control method. The results of these studies will be informative as efforts to attain an effective ASD disease control strategy continue for use across climatic conditions and soil pathogen profiles in various strawberry growing regions and develop a comprehensive understanding of underlying functional mechanisms contributing to disease control.

## Figures and Tables

**Table 1:** Summary of experimental variables employed in the four anaerobic soil disinfestation (ASD) trials conducted in this study.

Experiment	Soil	^a^ Treatments	^b^ C-Sources	Rate	Incubation Temperature (Day/Night)	Incubation Duration	Pathogen
Incubation temperature and duration study	Strawberry field soil, Watsonville, California	ASD-non-pasteurized soil with a carbon input NTC-non-pasteurized aerobic soil without a carbon input	RB	*20 t ha^−1^*	Low (L)-16/10 °C Medium low (ML)-24/18 °C Medium (M)-32/26 °C High (H)-40/34 °C	3 or 6 weeks	*F. oxysporum* f. sp. *fragariae* and *M. phaseolina* (natural infestation)
‘Standard rate’ Gramineae carbon source study	SR soil, Rock Island, Washington	ASD-non-pasteurized soil with a carbon input NTC-non-pasteurized aerobic soil without a carbon input	GR WH RB	*20 t ha^−1^* *20 t ha^−1^* *20 t ha^−1^*	24/18 °C	3 weeks	*M. phaseolina* (artificial infestation)
‘Low rate trial 1′ Gramineae carbon source study	CV soil, Orondo, Washington	ASD-non-pasteurized soil with or without a carbon input NTC-non-pasteurized aerobic soil without a carbon input	GR RB	*10 t ha^−1^* *5.0 t ha^−1^*	24/18 °C	3 weeks	*F. oxysporum* f. sp. *fragariae* (artificial infestation)
Metabolome and microbiome and ‘Low rate trial 2′ Graminae carbon source studies	CV soil, Orondo, Washington	ASD-non-pasteurized soil with a carbon input ASD-NA-no-amendment anaerobic control non-pasteurized soil	GR WH RB	*10 t ha^−1^* *10 t ha^−1^* *5.0 t ha^−1^*	24/18 °C	3 weeks	*F. oxysporum* f. sp. *fragariae* (artificial infestation)

^a^ Treatments: ASD = anaerobic soil disinfestation; ASD-NA = no-amendment ASD control; NTC = no-treatment control. ^b^ C-sources: GR = ground orchard grass (*Dactylis glomerata* L.); WH = ground wheat grass (*Triticum aestivum* L. cv. ‘Alpowa’); RB = rice bran.

**Table 2:** Two-way analysis of similarity (ANOSIM) results comparing communities of carbon-amended soils to no-amendment controls.

			ASD Carbon Source					
			Orchard Grass ^a^		Wheatgrass		Rice Bran	
			Day	Treatment	Day	Treatment	Day	Treatment
Bacterial OTUs		*R* ^b^	0.56866	0.98413	0.70118	1	0.61451	1
		*p*	0.0001	0.0001	0.0001	0.0001	0.0001	0.0001
Fungal OTUs		*R*	0.38297	0.8254	0.36835	0.65608	0.35047	0.73016
		*p*	0.0001	0.0001	0.0001	0.0001	0.0001	0.0001
Volatile metabolites		*R*	0.55761	0.57407	0.52099	0.8642	0.88971	0.78395
		*p*	0.0001	0.0002	0.0001	0.0001	0.0001	0.0001
Polar metabolites		*R*	0.37048	0.81893	0.30647	0.81481	0.28498	0.57202
		*p*	0.0001	0.0001	0.0001	0.0001	0.0001	0.0001

^a^ Similarity of bacterial OTUs, fungal OTUs, volatile metabolites, and polar metabolites of ASD soils amended with a carbon source (orchard grass 10 t ha^−1^; wheatgrass 10 t ha^−1^; rice bran 5.0 t ha^−1^) were compared to ASD soils conducted without the amendment (ASD-NA). ^b^ Analysis of similarity was conducted using the Bray–Curtis similarity coefficient and permutation N = 10,000. *p* values < 0.05 were considered significant.

**Table 3:** Effect of anaerobic soil disinfestation carbon source on relative strawberry crown infection (%) by *Macrophomina phaseolina* and quantity of pathogen DNA (pg g^−1^) detected in crown tissue in trials 1 and 2.

Parameter	Soil Treatment ^a^				
	ASD-GR	ASD-RB ^b^	ASD-WH	NTC	PC
trial 1					
Percent crown infection	70.0	NA	36.8	59.6	63.2
*M. phaseolina* DNA in crown (×1000 pg g^−1^)	49 a	NA	230 a	420 a	440 a
trial 2					
Percent crown infection	30.4	72	0	44.4	0
*M. phaseolina* DNA in crown (×1000 pg g^−1^)	91.1 b	45.4 a	0.00094 b	32 a	1.002 b

^a^ ASD-GR = Anaerobic Soil Disinfestation (ASD) with grass residues applied at 20 t ha^−1^ rate; ASD-RB = ASD with rice bran applied at 20 t ha^−1^; ASD-WH = ASD with wheat residues applied at 20 t ha^−1^; NTC = no-treatment control; PC = pasteurized control treatment, and NA = Data not available. Values represent percentage of crown infection and *M. phaseolina* DNA in crown across all replicates with *n* = 10. Means designated with the same letter are not significantly different (*p* < 0.05). ^b^ ASD-RB treatment was not included in trial 1.

## Data Availability

The data presented in this study are available in the current article and in the Supplementary Material (S1–S3).

## Supplementary Materials

The following are available online at https://www.mdpi.com/article/10.3390/microorganisms9081638/s1, Table S1: Effect of treatment on plant fresh weight (g) at harvest for strawberry planted in *Fusarium oxysporum* f. sp. *fragariae* and *Macrophomina phaseolina* infested soils, respectively; Figure S1. Relative similarity of fungal communities during a simulated anaerobic soil disinfestation (ASD) treatment conducted using grass (A), wheat (B) or rice bran (C) as the carbon source compared to ASD conducted without the amendment (ASD-NA); Figure S2. Effect of soil treatment on fresh root biomass and fresh total biomass of strawberry plants at harvest in trial 2; Figure S3. Effect of soil treatment, incubation temperature, and duration on (A) *Macrophomina phaseolina* crown infection percentage in trial 2 (B) *Fusarium oxysporum* f. sp. *fragariae* crown infection percentage in trial 2, Supplementary Material_Soil Metabolome Data_S2, Supplementary Material_Soil Microbiome Data_S3.
