# Supplementary material for: Analysis of Environmental Variables and Carbon Input on Soil Microbiome, Metabolome and Disease Control Efficacy in Strawberry Attributable to Anaerobic Soil Disinfestation
Source: Microorganisms. 2021 Jul 31;9(8):1638. doi: 10.3390/microorganisms9081638 (PMC8401821; doi:10.3390/microorganisms9081638)
Supplement: Supplementary file 1 [file microorganisms-09-01638-s001.zip › Hewavitharana et al. 2021.Supplementary Materials_S1.pdf]

Hewavitharana, S.S.; Klarer, E.; Muramoto, J.; Shennan, C.; Mazzola, M. Analysis of Environmental Variables and Carbon Input on Soil Microbiome, Metabolome and Disease Control Efficacy in Strawberry Attributable to Anaerobic Soil Disinfestation. *Microorganisms* **2021**, *9*, 1638. https://doi.org/10.3390/microorganisms9081638

Supplementary Material

TABLE S1. Effect of treatment on plant fresh weight (g) at harvest for strawberry planted in *Fusarium oxysporum* f. sp. *fragariae* and *Macrophomina phaseolina* infested soils respectively.

Treatment[a]	*Fusarium oxysporum* f. sp. *fragariae* Infested soil		*Macrophomina phaseolina* infested soil	
	Trial 1	Trial 2	Trial 1	Trial 2
ASD	7.44 ± 0.50 a	21.29 ± 1.26 a	9.22 ± 0.46 a	21.63 ± 0.83 a
NTC	3.82 ± 0.50 b	7.70 ± 1.41 b	7.74 ± 0.46 b	14.01 ± 0.83 b

[a]Treatment: ASD =Anaerobic Soil Disinfestation; NTC = no treatment control. Values represent mean fresh biomass at harvest across all replicates with n = 32. Error values represent standard error of the mean. Means designated with the same letter are not significantly different ($P < 0.05$) as determined by Fisher's least significant difference method.

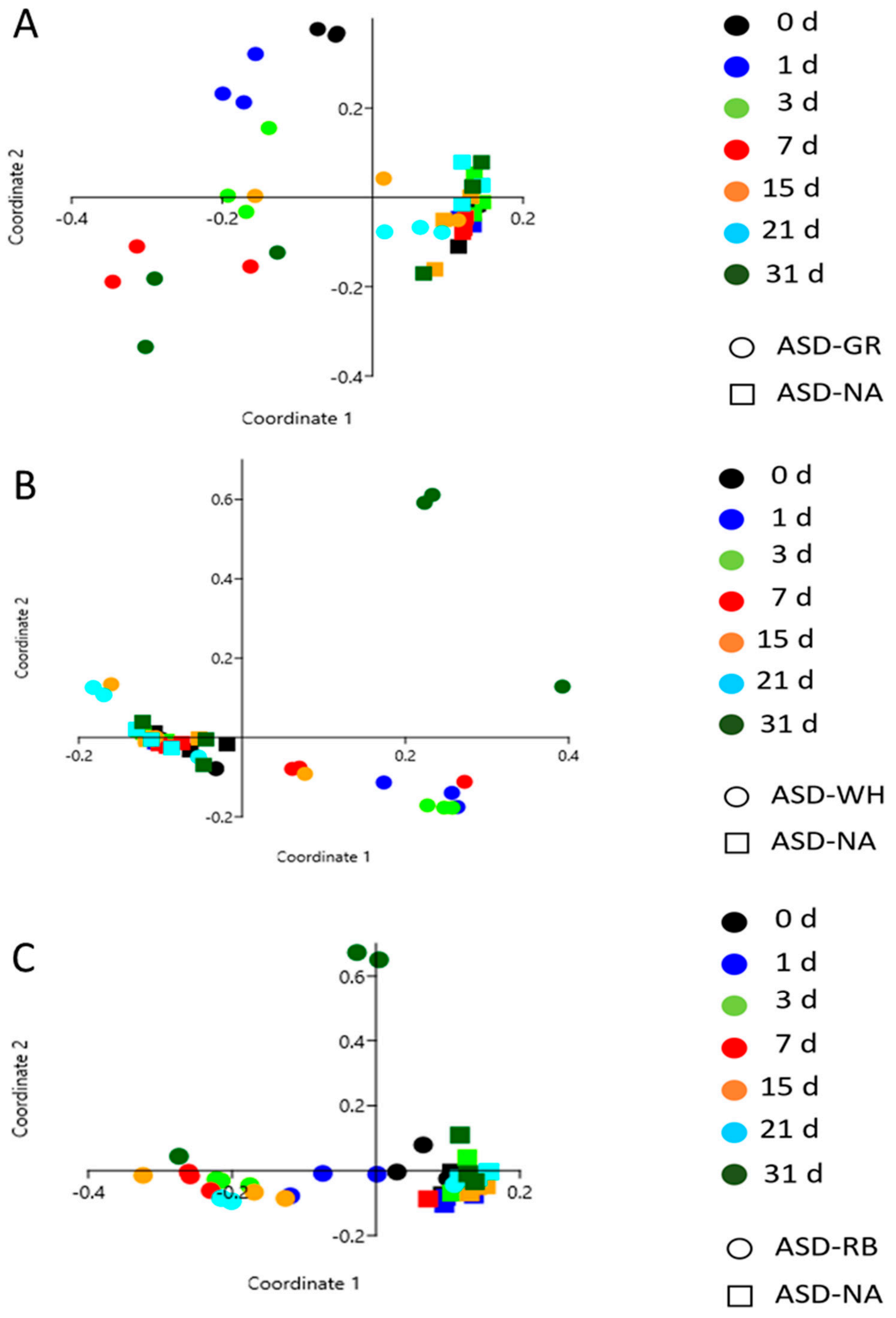

Figure S1. Relative similarity of fungal communities during a simulated anaerobic soil disinfestation (ASD) treatment conducted using grass (A), wheat (B) or rice bran (C) as the carbon source compared to ASD conducted without the amendment (ASD-NA). Ordination of soil microbiomes was conducted by principal coordinates analysis of operational taxonomic unit data using the Bray-Curtis similarity coefficient.

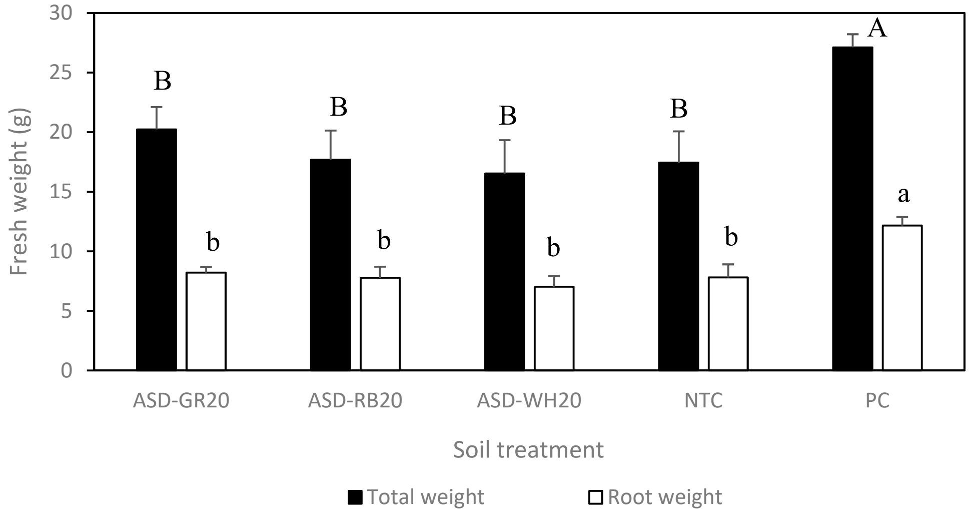

Figure S2. Effect of anaerobic soil disinfestation carbon source on fresh root biomass and fresh total biomass of strawberry plants at harvest in trial 2. Soil treatments included anaerobic soil disinfestation (ASD) with carbon input of grass residues at 20 t ha-1 (ASD-GR), rice bran at 20 t ha-1 (ASD-RB), wheat residues at 20 t ha-1 (ASD-WH), no treatment control (NTC) and pasteurized control treatment (PC). Values represent mean total plant biomass or root biomass across all replicates with n = 10. Error bars represent standard error of the mean. Means designated with the same letter are not significantly different ($P < 0.05$) and determined by Fisher's least significant difference method.

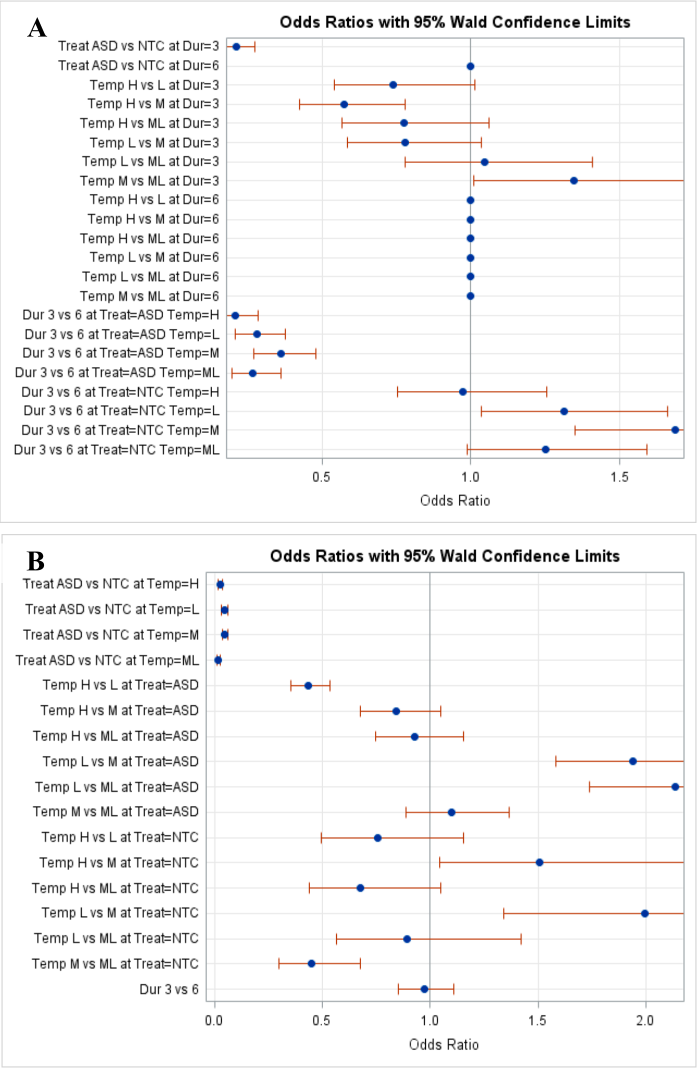

Figure S3. Effect of anerobic soil disinfestation carbon source, incubation temperature, and duration on (A) *Macrophomina phaseolina* crown infection percentage and (B) *Fusarium oxysporum* f. sp. *fragariae* crown infection percentage in trial 2. Soil treatments were Anaerobic Soil Disinfestation (ASD) and no treatment control (NTC). Day/night soil incubation temperature regimes (Temp) were L (low-16/10 °C), ML (medium low-24/18 °C), M (medium-32/26 °C), and H (high-40/34 °C). Durations (Dur) were 3 and 6 weeks. Odds Ratio (OR) equal to 1 indicates that a particular factor does not affect crown infection percentage. OR > 1 indicates the factor is associated with higher odds of outcome. OR < 1 indicates that the factor is associated with low odds of outcome.
